# The Effect of Silica Fume and GGBFS as Cement Substitutes on the Mechanical Properties and Water Resistance of Uncalcined Desulfurization Gypsum-Based Composite Cementitious Materials

**DOI:** 10.3390/ma19194077

**Published:** 2026-09-23

**Authors:** Siwei Zeng, Jiale Yan, Duotian Xia

**Affiliations:** 1 College of Water Conservancy & Architectural Engineering, Shihezi University, Shihezi 832003, China; zsw_shz@163.com (S.Z.); 15689432032@163.com (J.Y.); 2 Xinjiang Production & Construction Corps Key Laboratory of Civil Engineering Structural Safety in Cold, Arid and Strong Earthquake Regions, Shihezi 832003, China

**Keywords:** uncalcined desulfurization gypsum, silica fume, ground granulated blast furnace slag, mechanical properties, water resistance, microstructures

## Abstract

This study aims to reduce cement content and enhance mechanical and water resistance properties of uncalcined desulfurization gypsum-based composite cementitious materials (UDGCMs) by incorporating ground granulated blast furnace slag (GGBFS) and silica fume (SF) as cement replacements. The findings indicate that moderate incorporation of either SF or GGBFS optimizes the mechanical properties and water resistance of UDGCMs. Adding SF alone yields the greatest improvement in mechanical properties and markedly reduces the relative embodied energy, carbon emissions, and cost of UDGCMs. Adding GGBFS alone has a more pronounced effect in enhancing water resistance, and the softening coefficient can be increased by 30.98%. Under cement-free conditions, the blend of GGBFS and SF activated by a weakly alkaline environment provided by quicklime yielded a maximum 28-day compressive strength of 25.48 MPa. Microstructural analysis reveals that, in systems with separate additions of SF or GGBFS, strength enhancement is primarily achieved by modulating the formation of ettringite (AFt) and C-(A)-S-H gel. Under Portland cement-free conditions with simultaneous incorporation of SF and GGBFS, although the extent of the pozzolanic reaction is limited, the synergistic accumulation of ettringite and gel products can further promote material strength. This research provides a theoretical and experimental basis for uncalcined desulfurization gypsum (UDG) application and advances low-carbon cementitious materials.

## 1. Introduction

Over the past few years, the transition to green and low-carbon in industry and construction has become one of the most important developmental needs. Cement, a basic construction material crucial to national industrialization and urbanization, continues to rise in demand owing to population growth and rapid urbanization [1]. According to statistics, global cement production reached 4.16 × 10^9^ tons in 2022, accounting for 6–8% of CO_2_ emissions and contributing to the release of nitrogen oxides, SO_3_, and other harmful gases, thereby exacerbating the greenhouse effect and acid rain issues [1,2,3]. Owing to its high energy consumption and substantial environmental footprint, cement production poses a significant challenge to achieving sustainable development objectives [4]. Meanwhile, using industrial solid waste provides a viable means to address resource shortages and mitigate environmental impacts. Desulfurization gypsum (DG), an industrial by-product generated from coal-fired power plants that adopt wet flue gas desulfurization processes, is composed predominantly of calcium sulfate dihydrate (CaSO_4_·2H_2_O) [5]. Its primary application lies in the production of building materials [6], with additional uses as a cement set retarder [7], a subgrade backfill material [8], and an ameliorant for saline-alkali soils [9,10]. Despite a global increase in the total utilization of DG, its overall utilization rate remains relatively low due to the growing volume of DG production [11]. Large quantities of DG are stockpiled, occupying valuable land resources and posing risks of secondary environmental pollution [12]. Consequently, the development of low-carbon composite cementitious materials derived from DG is essential not only for improving solid waste utilization but also for advancing a low-carbon circular economy.

DG exhibits advantages including low impurity content and uniform particle size. However, its low strength and poor water resistance limit its large-scale practical application [6,13]. To enhance the strength and water resistance of DG, researchers developed a composite cementitious system by calcining DG and investigated the modification mechanisms of multi-component materials on DG [14,15,16,17,18,19]. This approach, however, requires heating DG to 145–185 °C to convert its primary component into β-type hemihydrate gypsum [13]. In contrast, the direct use of UDG avoids the energy losses associated with calcination and presents a promising route for eco-friendly cementitious material development. Zhong et al. [20] reported a 28-day compressive strength of 22.6 MPa for a cementitious ternary binder whose formulation was 1:3:1 (UDG:fly ash (FA):GGBFS). Microstructural characterization further showed that large amounts of AFt and C–S–H gel dominated the hydration products. Similarly, Lei et al. [21] investigated a composite material incorporating a high dosage (60%) of UDG. After 22 h of steam curing at 85 °C followed by 28 days of standard curing, the resulting composite achieved a compressive strength exceeding 42 MPa and a softening coefficient of 0.93. Microstructural analysis further revealed that the incorporation of a high dosage of UDG provides a sufficient sulfate supply, which governs the formation and transformation of AFt and AFm. Furthermore, in steel slag-based cementitious materials, the activation effect of sulfates added from UDG promotes the hydration of silicates in both the cement and the steel slag, facilitates the growth of AFt, and optimizes the pore structure [22,23]. Notably, the improvements in mechanical properties and water resistance in these UDGCMs largely depended on the addition of cement. Currently, there remains a lack of research on further reducing the cement content while maintaining a high proportion of UDG and simultaneously improving the mechanical properties and water resistance of UDGCMs. Consequently, exploring the utilization of industrial solid waste as a partial cement replacement has become a critical research direction. In this context, SF and GGBFS are promising candidates due to their pozzolanic activity, which enables them to react in alkaline environments to form gels and other hydration products [24,25,26,27,28].

It is noteworthy that both SF and GGBFS are common industrial by-products. SF is primarily composed of silicon dioxide, whereas GGBFS contains silicon dioxide, calcium oxide, and aluminum oxide [29]. Earlier studies have indicated that incorporating appropriate proportions of GGBFS and SF can improve the water resistance and mechanical strength of DG-based materials. For example, Li et al. [30] demonstrated that incorporating 50 nm nano-silica with a mass fraction of 0.4% into calcined DG markedly enhances its water resistance and mechanical performance. Cui et al. [31] investigated on the effects of adding various admixtures on the mechanical and thermal insulation properties of desulfurized gypsum-based composite cementitious materials and found that incorporating 35% SF could increase the compressive strength to 34.17 MPa. In this system, cement and quicklime jointly provide an alkaline environment that triggers the pozzolanic reaction of the SF, thereby fully leveraging its role in enhancing mechanical properties. Wang et al. [14] demonstrated that adding an appropriate amount of GGBFS can further enhance the compressive strength of desulfurized gypsum-based composite cementitious materials. Microstructural analysis indicates that, under alkaline conditions and in the presence of sulfates, the GGBFS dissolves and releases large amounts of highly reactive amorphous SiO_2_ and Al_2_O_3_, leading to the formation of substantial amounts of C-S-H gel and AFt. Gou et al. [19] studied calcined DG–cement–GGBFS composites and reported that 15–30% GGBFS addition significantly enhanced both mechanical properties and water resistance. Despite these advances, current research remains limited in two key aspects. First, most studies focus on calcined desulfurization gypsum-based composite cementitious materials, whereas relatively few investigations have examined the effects of GGBFS and SF on UDGCMs, especially under conditions of high UDG proportion. Second, whereas the application of SF and GGBFS in modifying desulfurization gypsum-based cementitious materials relies on the alkaline environment provided by cement, the synergistic hydration mechanism between these two additives remains to be clarified in a cement-free system with a high UDG loading, where calcium oxide serves as the sole alkaline activator. Addressing these gaps is crucial for advancing the development of high-performance, low-carbon UDGCMs.

Against this background, the present study investigates high-UDG-content UDGCM systems in which cement is partially replaced by varying dosages of GGBFS and SF. This work aims to reduce cement consumption and develop high-performance, eco-friendly UDGCMs. Various proportions of SF and GGBFS were tested as cement substitutes to evaluate their effects on the mechanical properties and water resistance of UDGCMs. The compressive strength, flexural strength, softening coefficient, and water absorption rate were tested for a thorough evaluation of the material performance. Furthermore, the microstructure of UDGCMs was analyzed using thermogravimetric and derivative thermogravimetric analysis (TG-DTG), scanning electron microscopy (SEM), and X-ray diffraction (XRD) to elucidate the mechanisms underlying the observed improvements in mechanical property and water resistance.

## 2. Experiments

### 2.1. Raw Material

UDG primarily consists of CaSO_4_·2H_2_O and appears as a white powder, while FA is a light yellow powder. Both materials were obtained from Yuelongda Co., Ltd. in Shihezi, Xinjiang, China. Quicklime is a white powder with a CaO content of 93.59%. The Portland cement used was P.O.42.5, conforming to the relevant Chinese standard. SF occurs as blue-black granular particles and exists in agglomerated clusters instead of discrete primary nanoparticles. Therefore, its filler effect and pozzolanic reactivity differ to some extent from those of discrete primary nanoparticles. GGBFS is of S95-grade, presenting as a grayish-white powder. The chemical composition and particle size distribution of the raw materials are summarized in Table 1 and Figure 1. The median particle sizes of UDG, FA, quicklime, cement, GGBFS, and SF were 40.989 μm, 9.593 μm, 7.408 μm, 15.232 μm, 12.098 μm, and 7.166 μm, respectively.

Figure 2 illustrates the microscopic morphologies of the experimental raw materials utilized in the experiment. The UDG particles display plate-like morphology with flat, well-defined facets arranged in a petal-like fashion, which produces a porous microstructure. FA particles are spherical, with varying sizes and smooth surfaces, and a small amount of impurities adheres to their surfaces. Cement and quicklime particles exhibit irregular shapes. GGBFS is observed in irregular block formations. SF is characterized by short elliptical strips with small edges and rough surfaces.

### 2.2. Preparation and Curing of Specimens

The UDGCM is composed of UDG, FA, quicklime, cement, GGBFS, and SF. The matrix consists of 60% UDG and 40% FA by mass. All other admixture percentages are calculated based on the mass of the UDG-FA matrix. Relative to the total mass of the matrix, the remaining components are added at the following dosages: 6% quicklime, 10% total dosage for the combined blend of cement, GGBFS, and SF, and 25% mixing water. For each mixture, within the fixed total admixture proportion of 10%, the cement, GGBFS, and SF are proportioned accordingly, where GGBFS or SF partially replaces cement in that mixture. The proportioning ratios of the UDGCM mixtures are detailed in Table 2. The preparation process involved the following steps: 1. All raw materials were combined in a planetary cement mortar mixer according to the specified proportions and mixed thoroughly for 2 min. 2. Water was added to the planetary cementitious sand mixer, after which mixing was conducted for 1 min at lower speed of 300 r/min and then for 1 min at higher speed of 850 r/min. 3. The mixture was cast into a mold and then vibrated to achieve the desired shape. 4. After 24 h, the samples were demolded. 5. The demolded specimens were placed in a standard curing chamber at a temperature of 20 ± 2 °C and a relative humidity of >95% and maintained until the target curing age was reached. The experimental procedure is visually depicted in Figure 3.

### 2.3. Experimental Methods

#### 2.3.1. Mechanical Property Test

Specimens (40 mm × 40 mm × 160 mm) for mechanical property tests, cured to designated ages (7 days, 14 days, and 28 days), were tested using 300 kN and 200 kN universal testing machines. The mechanical properties were assessed in compliance with the Chinese standard GB/T 9776-2022 [32]. Three replicate specimens were prepared for each mix proportion for flexural strength tests. The flexural strength was gauged by testing and calculating the arithmetic mean of three individual sample results, with results reported to an accuracy of 0.01 MPa. For the compression test, six half-specimens were obtained from the fractured flexural specimens corresponding to each mix proportion. The broken specimens were then loaded at a rate of 2.4 kN/s until fracture. During compression testing, the fractured cross-section after flexural failure was adopted as the loaded surface. Specimens were placed centrally for vertical axial loading, and the ultimate compressive strength was averaged from six valid test results, accurate to 0.01 MPa.

#### 2.3.2. Water Resistance Test

Independent batches of specimens were prepared for this water-resistance test. Accordingly, the measured dry and wet compressive strengths differ from the 28 d standard-cured compressive strength reported in Section 2.3.1. Two sets of specimens with the same mix proportion, cured for 28 days, were initially dried together in an oven at 40 ± 2 °C to constant weight (i.e., the relative mass change between two successive measurements taken 4 h apart did not exceed 0.1%.). Each set contained three replicate 40 mm × 40 mm × 160 mm prismatic specimens. The dry and wet compressive strength testing followed the identical loading procedure described in the mechanical property section, yielding six fractured half-specimens for averaging per set. One group was then used to measure the dry compressive strength, while the second set was immersed in water at 20 ± 2 °C until constant weight (i.e., the relative mass change between two successive measurements taken 4 h apart did not exceed 0.1%), following which the wet compressive strength was determined. The softening coefficient (K) was derived using Equation (1).

(1) $$\begin{array}{c} K=\frac{R_{\mathrm{wet}}}{R_{\mathrm{dry}}} \end{array}$$

where K represents the softening coefficient, R_wet_ is the wet compressive strength, and R_dry_ is the dry compressive strength.

#### 2.3.3. Water Absorption

The water absorption was measured following the Chinese standard GB/T 9776-2022 for “Building gypsum”. The test blocks cured under standard conditions for 28 days were used. Three replicate 100 mm × 100 mm × 100 mm test blocks were prepared for each mix proportion. The test blocks were dehydrated in an oven at 40 ± 2 °C to a constant weight for weighing (M1). Subsequently, the blocks were submerged in water at room temperature for 24 h. Upon removal, excess surface water was wiped off, and the blocks were reweighed (M2). The water absorption rate was then calculated as (M2 − M1)/M1 × 100%. The final water-absorption result was the average value of three replicate specimens.

#### 2.3.4. Microstructure Characterization

To stop the alkali-activation reaction, the specimens cured under standard conditions for 28 days were subjected to a 24 h anhydrous ethanol immersion. Subsequently, the specimens were oven-dried to constant mass. After drying, a part of the material was pulverized and sieved with a 200-mesh sieve for later examination.

SEM: Microstructural observation was examined with a ZEISS Sigma 300 scanning electron microscope manufactured in Jena, Germany. Before observation, the specimens were cut into small pieces with a size not exceeding 5 mm. The pieces were then gold-sputtered and examined under secondary electron mode.

XRD: Phase identification by XRD was performed using a Bruker D8 Advance diffractometer manufactured in Karlsruhe, Germany. The measurement employed Cu Kα radiation, covering an angular interval from 5° to 90° (2θ), and a scan rate of 5°/min was applied. Vertical offsets were applied to XRD patterns, with a unified *y*-axis scale for both subgraphs during plotting. Raw diffraction data (2θ positions and intensities) were unchanged.

TG-DTG: Thermal behavior was evaluated using an SDT650 simultaneous thermal analyzer manufactured in New Castle, Delaware, America. A powder sample of about 20 mg was heated in a nitrogen atmosphere from 30 to 1000 °C at a ramp of 10 °C min^−1^.

## 3. Results and Discussion

### 3.1. Mechanical Property

#### 3.1.1. Compressive Strength

Figure 4 illustrates the effect of incorporating GGBFS and SF on the compressive strength of UDGCMs. Among the tested mixtures, C4-G6, C2-S8, and G2-S8 exhibited the most favorable performance in the C-G, C-S, and G-S systems, respectively, with C2-S8 achieving the highest compressive strength after 28 days of curing. In systems where GGBFS replaces cement, the 28-day compressive strength exhibits an initial increase followed by a decrease as the GGBFS content rises. The UDGCM (4% cement and 6% GGBFS) achieved a maximum compressive strength of 20.05 MPa at 28 days, representing a 16.64% increase compared to the reference group. This improvement is primarily attributed to the reaction between appropriate GGBFS and Ca^2+^ and SO_4_^2−^ dissolved from UDG, together with synergistic interactions with FA to promote the formation of C-(A)-S-H gel, which further increases the compressive strength of the material [22,33]. However, when GGBFS completely replaces cement, excessively high GGBFS dosage may induce pore enlargement as speculated from SEM images, and insufficient filling by gel and AFt could then yield a relatively loose matrix and lower mechanical strength [34].

SF plays a pronounced role in enhancing the compressive strength of UDGCMs. As the replacement ratio of SF increases, the strength at all curing ages shows a trend of rapid initial rise followed by a decline. The UDGCM (2% cement and 8% SF) achieved a maximum compressive strength of 29.95 MPa at 28 days, representing a 74.28% improvement compared to the reference group. This is primarily attributed to the highly reactive SiO_2_ in SF, which rapidly participates in reactions, generating a substantial amount of C-(A)-S-H gel, leading to structural densification and consequently enhancing the compressive strength [35,36]. However, when SF completely replaces cement, excessive SF covers the surface of UDG, forming a gel during hydration that encapsulates unreacted UDG, inhibiting its further dissolution and Ca^2+^ release [19]. This results in a decreased Ca^2+^ content within the system, failing to meet the Ca^2+^ demand during the hydration reaction, which reduces gel production and consequently leads to a decline in the compressive strength of the UDGCM. According to the literature [20], the ternary cementitious system composed of UDG, FA, and GGBFS achieves a compressive strength of 22.6 MPa after 28 days of standard curing. In comparison, the optimal compressive strength of UDGCM in this study reaches 29.95 MPa under 28-day standard curing. Under identical curing conditions, UDGCM not only exhibits certain strength advantages but also features a higher dosage of UDG within the system.

In a cement-free system where both SF and GGBFS are added, the weakly alkaline environment created by quicklime becomes the key driver for the hydration of GGBFS and SF. The UDGCM (2% GGBFS and 8% SF) achieved a maximum compressive strength of 25.48 MPa at 28 days, representing a 48.25% increase compared to the reference group. This indicates that even under Portland cement-free conditions, the system, through the combined incorporation of GGBFS and SF, retains good potential for compressive strength development. In this paper, the UDG not only serves as a source of Ca^2+^ and SO_4_^2−^, but its porous structure also facilitates the continuous progression of the pozzolanic reaction. SF exhibits a faster pozzolanic reaction rate compared to GGBFS [37]. Consequently, SF reacts preferentially to develop early-age strength, while GGBFS subsequently undergoes continued hydration in the later-stage alkaline environment, creating an effective complementary reaction sequence. Furthermore, the synergistic accumulation of rod-like AFt and C-(A)-S-H gel products among the generated hydration products can further fill pores and enhance the compactness of the UDGCM. However, constrained by the overall insufficient alkalinity of this system, the dissolution and reaction degree of GGBFS are limited [38], resulting in a lower content of hydration products, which is the primary reason its compressive strength does not surpass that of the highest-performing mix among the tested mixtures in the C-S system.

#### 3.1.2. Flexural Strength

Figure 5 illustrates the effect of incorporating GGBFS and SF on the flexural strength of the UDGCM. Flexural strength is highly sensitive to micro-cracks [39]. In systems where GGBFS replaces cement, the flexural strength fluctuates only within a limited range (amplitude < 0.5 MPa) as the GGBFS content increases, indicating that the incorporation of GGBFS exerts a limited impact on the flexural strength of UDGCMs. The UDGCM (4% cement and 6% GGBFS) achieved a maximum flexural strength of 4.32 MPa at 28 days, representing an 11.46% improvement over the reference group. It can be reasonably interpreted that C-(A)-S-H gel formed from the pozzolanic reaction of appropriate GGBFS can fill micro-cracks, thus potentially improving flexural strength to a certain degree [40]. However, when the GGBFS dosage is further increased, the flexural strength begins to decline. The primary reason is the reduced system alkalinity resulting from the decreased cement content, which limits the dissolution and hydration degree of GGBFS, leading to an insufficient crack-repairing capacity.

In systems where SF replaces cement, the flexural strength of specimens cured for 7 and 14 days initially increases and then decreases with the incorporation of SF. When the SF content is less than 6%, the rapid reaction of SF generates dense C-(A)-S-H gel, effectively filling pores and repairing early-stage micro-cracks, thereby enhancing the overall compactness and early-age flexural performance of the material [41]. However, the insufficient Ca^2+^ supply within the system results from the decreased cement content and the hindered dissolution of UDG once the SF content exceeds 6%. This restricts the continued formation of gel, thereby reducing its ability to fill micro-cracks and resulting in a decrease in flexural strength. As the curing age extends to 28 days, the flexural strength of this system exhibits fluctuation. This is because, in addition to the aforementioned influencing factors, the extended curing period combined with SF incorporation induces micro-cracks through internal restraint to autogenous shrinkage, thereby affecting the flexural strength of the UDGCM [42]. Therefore, the flexural strength of the single SF incorporation system is a result of multi-factor influences at 28 days.

In the cement-free system, the UDGCM (2% GGBFS and 8% SF) exhibits an excellent synergistic effect, enhancing flexural strength to a high level of 5.61 MPa at 28 days, representing a 44.81% increase compared to the reference group. SF first undergoes a rapid pozzolanic reaction, forming a dense structure at an early age. Subsequently, GGBFS hydrates slowly in the sustained weak alkaline environment provided by quicklime, generating C-(A)-S-H gel that fills the micro-cracks not fully repaired by the early reaction of SF, thereby further enhancing the flexural strength of the material.

A limitation of this study is that a fixed water dosage was adopted for all mixtures, and fresh-state workability was not characterized. Since cement, GGBFS and SF have large differences in specific surface area, changing their proportions can alter paste water demand, consistency and particle packing. These factors may in turn affect the measured mechanical performance.

### 3.2. Water Resistance

#### 3.2.1. Softening Coefficient

Figure 2a shows that raw UDG contains abundant inherent pores. These pores provide channels for moisture penetration and consequently result in poor water resistance of the composite. Nevertheless, their porous and permeable features, combined with the weakly alkaline environment supplied by quicklime upon SF and GGBFS addition, also provide essential space and moisture conditions for further pozzolanic reactions of these mineral admixtures after water ingress.

Figure 6 demonstrates the effect of incorporating GGBFS and SF on the softening coefficient of the UDGCM. When GGBFS replaced 60% of the cement fraction within the 10% binder component, the softening coefficient reached a peak value of 0.947, representing a 30.98% increase over the reference group. Additionally, the wet compressive strength also increased to 31.99 MPa, a 33.9% improvement. This improvement is primarily attributed to the appropriate GGBFS dosage under water-saturated conditions, which promotes secondary hydration reactions and generates C-(A)-S-H gel, thereby improving the wet compressive strength [34]. However, when the GGBFS content is further increased, an excessively high GGBFS content may cause pore coarsening within the matrix, thereby promoting water intrusion and leading to an overall decline in the softening coefficient.

Similarly, when SF replaced 6% of the cement, the softening coefficient increased to 0.920, representing a 27.25% increase. Moreover, its wet compressive strength reached the highest value among all mixes at 33.88 MPa, demonstrating the advantage of SF in enhancing the wet compressive strength of the material. This is mainly attributed to the high pozzolanic activity of SF, which promotes the formation of a stable gel [43]. Meanwhile, its micro-filling effect may help to fill the voids between UDG particles and hinder moisture penetration, thus increasing the softening coefficient [30]. It is noteworthy that, when SF completely replaced cement, the system lacked sufficient Ca^2+^ for sustained gel reactions, leading to a decrease in the densification of the material’s internal structure. In a cement-free system where SF and GGBFS are added together, the softening coefficient was maintained between 0.89 and 0.92, indicating that a cementitious system with good water resistance potential can still be constructed even without the addition of cement. In summary, utilizing the material’s inherent porous characteristics to guide beneficial hydration reactions is an effective pathway for achieving enhanced water resistance in UDGCMs.

#### 3.2.2. Water Absorption Rate

Figure 7 presents the effect of incorporating GGBFS and SF on the 24-h water absorption of the UDGCM. The curves reveal that, in systems with single incorporation of either GGBFS or SF, the water absorption initially decreases and then increases. Notably, for the C4-S6 mixture, the water absorption rate dropped to a minimum of 4.45%. In the cement-free system with the combined incorporation of GGBFS and SF, the water absorption showed a continuous decrease with increasing SF content. This indicates that, even in the cement-free, weakly alkaline environment, SF retains good dissolution and reactivity. The hydration gel it forms contributes to filling pores, thereby improving the compactness of the material. Combining the experimental results from Figure 6, it was found that, although the water absorption of mix C4-G6 was slightly higher than that of C4-S6, the softening coefficients of C4-G6 were superior to those of C4-S6. These macroscopic results lead rise to a reasonable speculative mechanism: penetrating water may sustain the pozzolanic reactions of GGBFS and SF, thereby promoting the formation of more gel and enhancing the repair and densification of the microstructure [44,45]. Therefore, water absorption is not the sole determinant of the softening coefficient. The sustained hydration capacity of SF and GGBFS under wet conditions plays a crucial role in the final water resistance of the material.

### 3.3. Microanalysis

#### 3.3.1. SEM Images Analysis

C4-G6, C2-S8, and G2-S8 are the optimal groups in C-G, C-S, and G-S, respectively. The macroscopic characteristics of the samples exhibit a strong correlation with their microscopic morphology. Figure 8 illustrates the microscopic morphology of various specimen groups at 28 days. Based on the clustered morphology, raw material composition, and the existing literature [31,46], this substance is speculated to be C-(A)-S-H gel.

Figure 8a illustrates that C10 displays minute cracks and a large amount of needle-rod shaped AFt crystals, with a scant presence of clustered C-S-H gel within its structure. Moreover, voids are evident among the matrix components, with some of these voids being filled with AFt crystals. Notably, certain FA particles exhibit smooth surfaces, indicating incomplete reaction of some FA particles in the specimen. This incomplete reaction likely plays a role in the insufficient generation of C-S-H gel, leading to inadequate overall density. These observations may suggest relatively low FA reactivity within C10, which could lead to insufficient gel formation and poorer compaction, and this may in turn compromise its macroscopic mechanical properties. During the hydration process of cement, AFt can fill the pores of the material to a certain extent and improve the strength, but excessive needle-rod shaped AFt is easy to squeeze in UDG internally and produce expansion stress, which leads to the generation of internal cracks in the material, and on the contrary, affects the overall mechanical properties of the material [47]. In Figure 8d, C4-G6 is observed to be extensively covered by small clusters of C-(A)-S-H gel, filling the pores over a significant area. This gel emerges as the predominant substance occupying the pores. Figure 8b illustrates that the hydration products of G10 consist of C-(A)-S-H gel and needle-and-rod AFt. The matrix contains relatively large pores with no fillers present. The presence of large unfilled pores in G10 may be associated with the high GGBFS dosage. It is speculated that excessive GGBFS incorporation could induce relatively high autogenous shrinkage, which may further enlarge matrix pores. Notably, these pores exhibit a deficiency of AFt and gel fillers. Pore imperfections can serve as critical sites of damage under stress, consequently impairing the mechanical properties of UDGCM [24].

In Figure 8e, cluster-like C-(A)-S-H and a small quantity of AFt are observed in C2-S8. The surface of the original FA particles is covered by hydration products, which signals the onset of the pozzolanic reaction, while low-polymerized gel remains attached to that surface. As the reaction progresses, the low-polymerized gel polymerizes into a gel adhering to FA, creating a dense structure and contributing to early strength [48,49,50]. Upon complete replacement of cement by SF, numerous micro-cracks develop in S10, as depicted in Figure 8c. Furthermore, the incorporated SF serves as nucleation sites within UDG, directing the UDG crystals to grow in a plate-like morphology and align closely to form a compact matrix structure [30]. Another portion of SiO_2_ adheres to the surface of UDG, undergoing a pozzolanic reaction that produces a significant amount of C-(A)-S-H gel encapsulating UDG. This process may hinder the dissolution and release of Ca^2+^ from UDG, thereby reducing gel formation in the reaction system [19]. A comparison of S10, C2-S8 with C10 revealed no large AFt crystals or large pores. This indicates that replacing cement with SF can efficiently promote gel formation, effectively filling pores and preventing the formation of large voids. By examining the microstructural morphology of C10, C2-S8, and S10, the impact of SF incorporation on the initial UDGCM is elucidated in terms of physical and chemical characteristics.

In Figure 8f, G2-S8 exhibits rod-like AFt crystals interlaced with one another, filling the gaps and forming a dense structure that substantially enhances the strength and water resistance of UDGCM [48,49]. Moreover, some rod-like AFt and gel products synergistically accumulate, further filling internal pores, which contributes to the higher strength of G2-S8 compared to G10. FA particles display irregular morphologies, with traces of incompletely hydrated surfaces, which may imply limited pozzolanic reactivity and an insufficient reaction degree. Although dual incorporation of GGBFS and SF without cement may result in incomplete FA reaction, the overall material density remains relatively high due to the synergistic accumulation of rod-like AFt and gel products. Combined with previous analyses of each FA particle group, it can be concluded that the addition of SF enhances FA consumption within the reaction system.

#### 3.3.2. TG-DTG Analysis

As shown in Figure 9, These samples exhibit ongoing mass reduction at distinct temperatures, encompassing primarily four weight loss zones. In the first stage, C-(A)-S-H and AFt decompose at 50–100 °C [51]. In the second stage, CaSO_4_·2H_2_O loses water at 100–180 °C [51,52]. In the third stage, calcium hydroxide decomposes at 400–500 °C [53]. In the fourth stage, carbonates decompose at 540–710 °C [54].

Table 3 presents the weight loss rates of C10, G10, S10, C4-G6, C2-S8, and G2-S8 across different temperature ranges. It should be noted that this approach has inherent limitations due to overlapping phase-decomposition ranges. Nevertheless, it allows reasonable approximate comparison of relative phase contents across different groups [18]. The weight loss values of C10, G10, S10, C4-G6, C2-S8, and G2-S8 at 50–100 °C are 3.05%, 1.84%, 2.03%, 1.23%, 2.83%, and 2.66%, respectively. Based on these relative weight-loss values, a speculative trend for the overall abundance of AFt and C-(A)-S-H may be inferred as: C10 > C2-S8 > G2-S8 > S10 > G10 > C4-G6. This tentatively suggests that cement substitution by GGBFS and SF may lower the overall abundance of hydration products in UDGCMs. Notably, as the SF dosage increases from 8% to 10%, the total content of AFt and C-(A)-S-H decreases, whereas the AFt content increases, indicating a reduction in C-(A)-S-H content. The weight losses of C10, G10, S10, C4-G6, C2-S8, and G2-S8 in the 100–180 °C range are 12.26%, 11.59%, 12.70%, 11.25%, 11.99%, and 11.83%, respectively. From these data, a presumed relative trend for CaSO_4_·2H_2_O can be observed: S10 > C10 > C2-S8 > G2-S8 > G10 > C4-G6. As the main component of UDG, CaSO_4_·2H_2_O content reflects the extent of UDG dissolution. Notably, S10 exhibits the highest content of undissolved CaSO_4_·2H_2_O, indicating that UDG dissolution is inhibited when the SF content reaches 10%. The weight loss values of C10, G10, S10, C4-G6, C2-S8, and G2-S8 at 400–500 °C are 1.15%, 0.97%, 0.56%, 1.15%, 0.62%, and 0.60%, respectively. C10 and C4-G6 exhibit the largest weight losses, whereas S10, C2-S8, and G2-S8 show smaller weight losses. This may tentatively suggest that SF addition could promote calcium hydroxide consumption within the system. The weight reductions of C10, G10, S10, C4-G6, C2-S8, and G2-S8 at 540–710 °C are 2.04%, 1.82%, 1.70%, 1.91%, 1.56%, and 1.80%, respectively. These differences may imply that samples S10, C2-S8, and G2-S8 have comparatively less carbonate formed via reaction between OH^—^ and atmospheric CO_2_. Combined with the above-mentioned trends, this provides further inferential support that SF addition may enhance calcium-hydroxide consumption in the reaction system.

#### 3.3.3. XRD Analysis

Figure 10 displays the XRD patterns of hydration products from assorted groups at 28 days. The predominant phases observed are CaSO_4_·2H_2_O and Aft. CaSO_4_·2H_2_O constitutes the primary component of UDG, while Aft is a hydration product.

The peak intensity of Aft (2θ 9.2°) gradually decreases with the increase in GGBFS replacement ratio of cement. As GGBFS incorporates more active aluminosilicate components into the system, abundant amorphous C-(A)-S-H gel tends to form and consumes dissolved Ca^2+^ and Al^3+^ in pore solution. The competitive consumption of reactive ions suppresses the precipitation of crystalline AFt, accompanied by the delayed consumption of dihydrate gypsum from UDG, leading to the first decline and subsequent rebound of CaSO_4_·2H_2_O diffraction intensity. It should be noted that the variation trend in mechanical strength of UDGCMs does not correspond to the relative change inferred from the AFt peak intensity. This may suggest that, when the GGBFS replacement ratio of cement reaches 6%, the dominant contributor to strength gain is not AFt, but rather the synergistic reaction between GGBFS and FA under alkaline conditions. This reaction may consume a significant amount of Ca^2+^ to form gel, which can improve the compactness of UDGCMs.

The phase intensities of AFt (2θ 9.2°) and CaSO_4_·2H_2_O (2θ 11.7°) decrease when 8% of the cement is replaced by SF. Notably, C2-S8 exhibits a diffuse hump in the range of 25–40°, signifying the presence of amorphous hydration products. These products are likely C-(A)-S-H gel [18]. When SF replaces 8% of cement, the SiO_2_ content in the reaction system increases, while the contents of tricalcium silicate (C_3_S), dicalcium silicate (C_2_S), and Al_2_O_3_ decrease. This shift promotes further dissolution of UDG, the primary calcium source, supplying additional Ca^2+^ and SO_4_^2−^ ions to the system. In the presence of adequate Ca^2+^ levels, hydration reactions are preferentially directed towards gel formation [55]. This provides a plausible mechanistic explanation for the diffuse hump signal observed in the XRD pattern of C2-S8. Conversely, an excessive dosage of SF results in hydration products adhering to the surface of UDG and hinders its complete dissolution [19]. This surplus of SF, coupled with limited Ca^2+^ availability, shifts the hydration pathway toward predominant crystalline AFt formation. It should be noted that the mass loss of TG-DTG in the range of 50–100 °C originates from the combined dehydration of crystalline AFt and amorphous C-(A)-S-H gel. By contrast, XRD can only identify crystalline phases. Amorphous C-(A)-S-H gel is characterized merely by a diffuse hump at 25–40° without sharp diffraction peaks. Based on the combined analysis of TG-DTG and XRD results, S10 exhibits higher diffraction-peak intensity of crystalline AFt but lower total mass loss over 50–100 °C compared with C2-S8. A prominent diffuse hump assigned to amorphous C-(A)-S-H gel occurs at 25–40° 2θ for C2-S8. This signal remains weak in S10. These combined observations further confirm that sufficient calcium ions in C2-S8 facilitate the generation of abundant amorphous C-(A)-S-H gel. Calcium-ion deficiency in S10 drives the preferential formation of crystalline AFt.

As the proportion of SF in the binary SF–GGBFS blend increases, the diffraction peak intensities of AFt (2θ = 9.2°) increase noticeably, whereas those of UDG remain relatively stable. This phenomenon implies that the inclusion of SF in the binary blend system facilitates the generation of AFt, which then occupies the voids within the material, thereby contributing to its enhanced strength. This observation accords with the discoveries from the microstructure’s SEM analysis.

On the basis of the XRD analysis, it was observed that C4-G6 and G2-S8 lacked dispersion peaks akin to those of C2-S8, indicating a higher production of C-(A)-S-H gel products by C2-S8. Further comparison of AFt (2θ 9.2°) peak intensities among G2-S8, C2-S8, and C4-G6 samples suggests, from a comprehensive perspective, that the relatively lower hydration extent of C4-G6 may be one factor responsible for its inferior mechanical performance.

### 3.4. Economic and Environmental Assessment

Reducing embodied energy, reducing carbon emissions, and constraining production costs are the principal drivers behind the advancement of green building practices. Substituting cement with SF and GGBFS reduces cement consumption while simultaneously delivering demonstrable advantages in embodied energy, CO_2_ emissions, and economic cost. Given that existing studies have shown that the raw material production stage contributes over 84% of the carbon footprint of concrete throughout its life cycle [56], this study focuses solely on the carbon emissions from the production of raw materials for UDGCM. This analysis represents a screening-level raw-material-oriented environmental–economic assessment rather than a complete full-life-cycle assessment.

Table 4 lists the carbon emission factors of raw materials during production. Equations (2)–(4) were used to calculate the embodied energy, carbon emissions, and cost of each mix proportion, respectively. Relative embodied energy, relative carbon emissions, and relative cost are introduced to evaluate the comprehensive utilization efficiency of the material. The relative embodied energy, relative carbon emissions, and relative cost are then obtained by dividing these values by the compressive strength of the corresponding mix proportion.

(2) $$\begin{array}{c} E_{x}=\frac{1}{1000}\sum E_{x,i}\times Q_{x,i} \end{array}$$

where E_x_ is the total embodied energy of each mix proportion, GJ/m^3^. E_x,i_ is the embodied energy factor of ith raw material, MJ/kg. Q_x,i_ is the usage of ith raw material, kg/m^3^.

(3) $$\begin{array}{c} C_{x}=\frac{1}{1000}\sum C_{x,i}\times Q_{x,i} \end{array}$$

where C_x_ is the total carbon emissions of each mix proportion, t CO_2_-eq/m^3^. C_x,i_ is the carbon emission factor of ith raw material, kg CO_2_-eq/kg. Q_x,i_ is the usage of ith raw material, kg/m^3^.

(4) $$\begin{array}{c} U_{x}=\sum U_{x,i}\times Q_{x,i} \end{array}$$

where U_x_ is the total cost of each mix proportion, USD/m^3^. U_x,i_ is the cost factor of ith raw material, USD/kg. Q_x,i_ is the usage of ith raw material, kg/m^3^.

Figure 11 presents a comparison of economic and sustainability indicators of UDGCM mixtures with different binder proportions. As shown, carbon emissions and embodied energy of UDGCMs are both significantly lowered by using SF and GGBFS instead of cement. However, owing to the higher unit cost of SF relative to cement, the S10 and C2-S8 mixtures register an increase of 20.54% and 16.44% in cost per kilogram of UDGCM, respectively, compared to the C10 control. The C2-S8 mixture exhibits the lowest relative values for embodied energy (48.67 MJ/m^3^·MPa), carbon emissions (13.44 kg CO_2_-eq/m^3^·MPa), and cost (2.14 USD/m^3^·MPa) among all formulations. It should be specially noted that, although C2-S8 has low relative embodied energy, relative carbon emissions, and relative cost, its absolute cost increases due to the high unit price of SF. This indicates that the C2-S8 mixture significantly enhances the compressive strength of UDGCM, sufficiently offsetting the economic disadvantage arising from the higher material cost of SF. In a Portland cement-free cementitious system, incorporating SF and GGBFS is an effective strategy for reducing relative embodied energy, relative carbon emissions, and relative cost. Specifically, compared with C10, the G2-S8 mixture exhibited reductions in relative energy consumption, relative carbon emissions, and relative cost by 55.84%, 50.36%, and 22.48%, respectively. Although S10 and G10 also achieved complete cement replacement, their lower compressive strength resulted in higher values for all three relative indicators than those of G2-S8. Notably, eliminating Portland cement cannot completely eliminate the environmental burden of UDGCMs, because quicklime, which serves as an alkaline activator, still has relatively high embodied energy and carbon emissions.

In conclusion, if the primary objective is to achieve a Portland cement-free UDGCM formulation, a complete substitution strategy employing 8% SF combined with 2% GGBFS is recommended. Conversely, when comprehensive environmental and economic performance is prioritized as the decisive criterion, then it is recommended to use 8% SF to partially replace cement.

## 4. Conclusions

(1) The single incorporation of SF demonstrates a particularly outstanding effect on enhancing compressive strength. Specifically, the UDGCM (2% cement and 8% SF) achieved a compressive strength of 29.95 MPa at 28 days, representing a 74.28% improvement over the reference group. However, when SF fully replaces Portland cement, the dissolution of UDG is suppressed, which reduces the available Ca^2+^ concentration and shifts the dominant hydration products from C-(A)-S-H gel to AFt.
(2) The single incorporation of GGBFS exhibits superior performance in enhancing water resistance. For the UDGCM (4% cement and 6% GGBFS), the softening coefficient reached 0.947. However, when the GGBFS dosage is further increased, insufficient hydration reduces the C-(A)-S-H gel content, thereby degrading the material performance.
(3) Under Portland cement-free conditions, the UDGCM (2% GGBFS and 8% SF) exhibited the best overall performance. After 28 days of curing, the compressive strength and flexural strength reached 25.48 MPa and 5.61 MPa.
(4) Microstructural analysis indicates that SF incorporation promotes the consumption of FA and Ca(OH)_2_, enhances the pozzolanic reaction, and induces a compact plate-like arrangement of UDG crystals. In the Portland cement-free system, despite the limited pozzolanic reaction when SF (8%) and GGBFS (2%) are blended, the synergistic accumulation of AFt and C-(A)-S-H gel can further improve material strength.
(5) Comparative economic and environmental assessment indicates that replacing cement with 8% SF alone offers the advantages of lower relative energy consumption, carbon emissions, and cost.

Despite the favorable mechanical and water-resistant performance of the developed UDGCM, several limitations remain to be addressed. This study mainly focuses on the 28-day macroscopic properties of UDGCMs, while long-term service performances, including dimensional stability, drying shrinkage, wet–dry cycle resistance, and long-term mechanical degradation, have not been systematically evaluated. In particular, as a composite rich in industrial by-products, the long-term environmental safety and leaching behavior of UDGCMs still require further verification. In terms of practical application, UDGCM with a strength range of 25–30 MPa is suitable for non-structural components such as precast blocks and road base filling. Nevertheless, further studies on durability optimization and environmental assessment are necessary to support its large-scale engineering promotion.

## Figures and Tables

**Figure 1:**
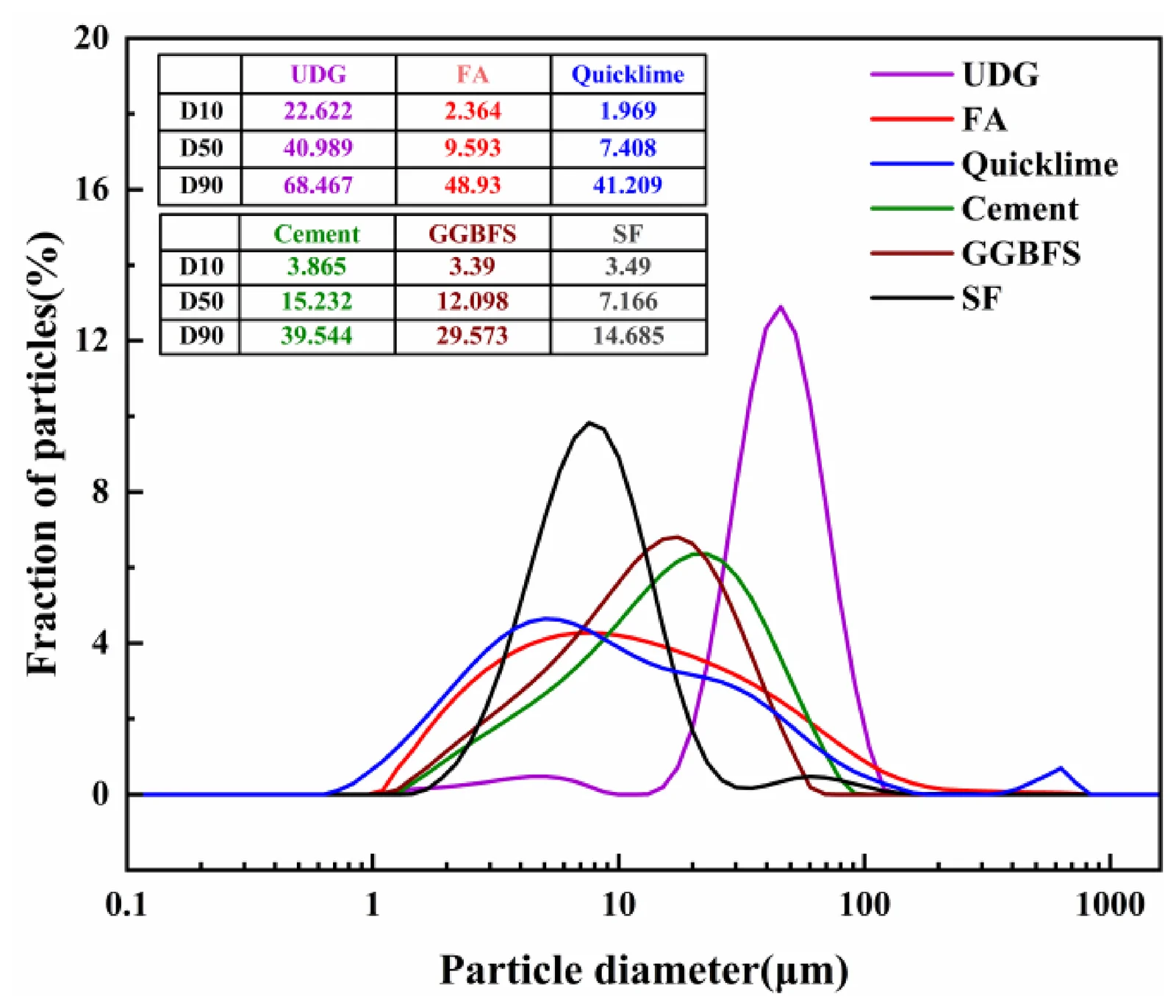
Particle size distribution curves of experimental raw materials.

**Figure 2:**
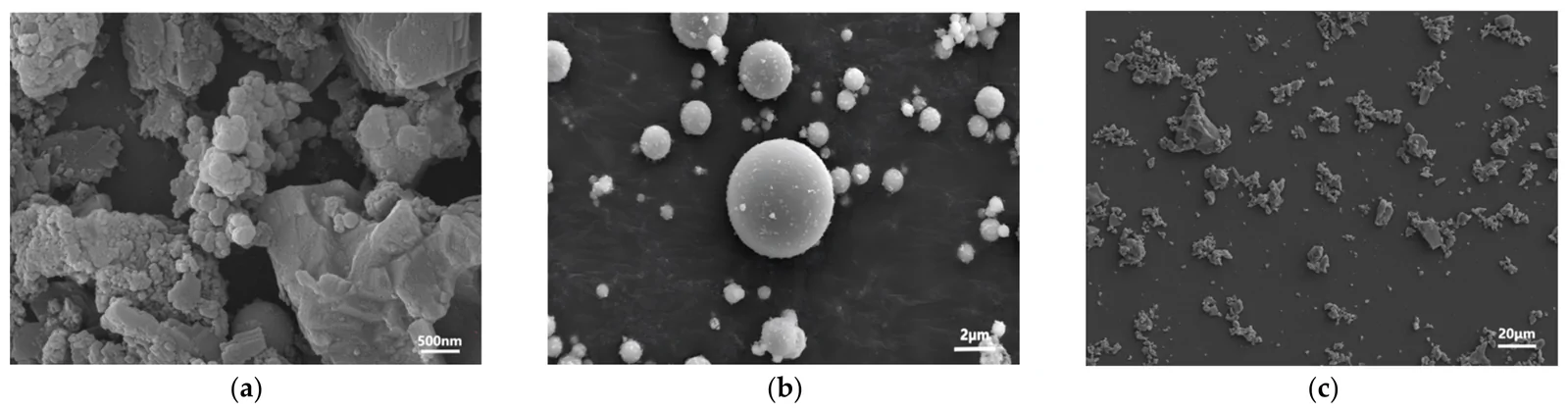

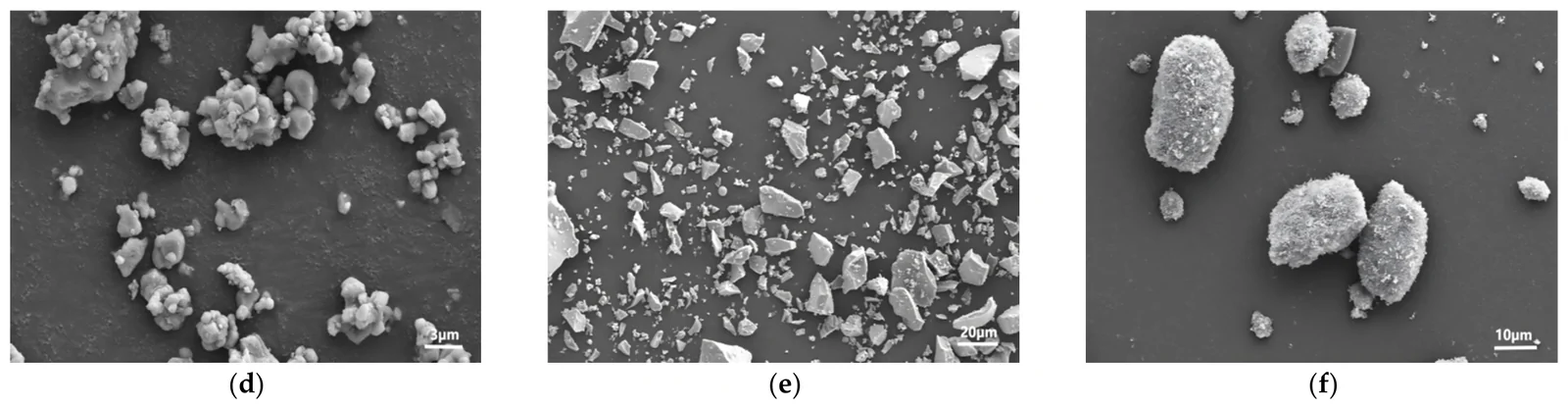
Microscopic morphologies of experimental raw materials. (**a**) UDG, (**b**) FA, (**c**) Cement, (**d**) Quicklime, (**e**) GGBFS, (**f**) SF.

**Figure 3:**
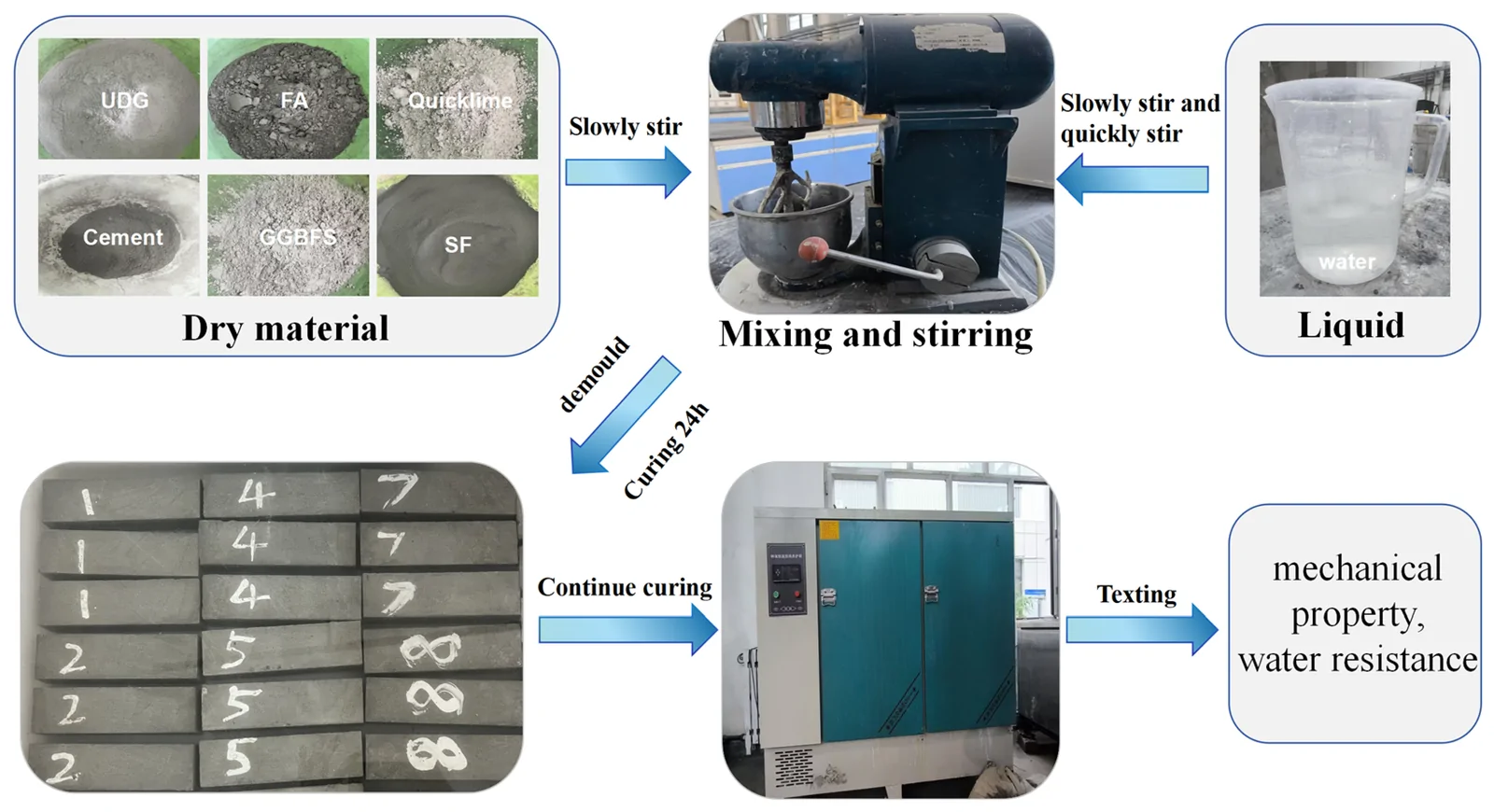
The preparation procedure of specimen.

**Figure 4:**
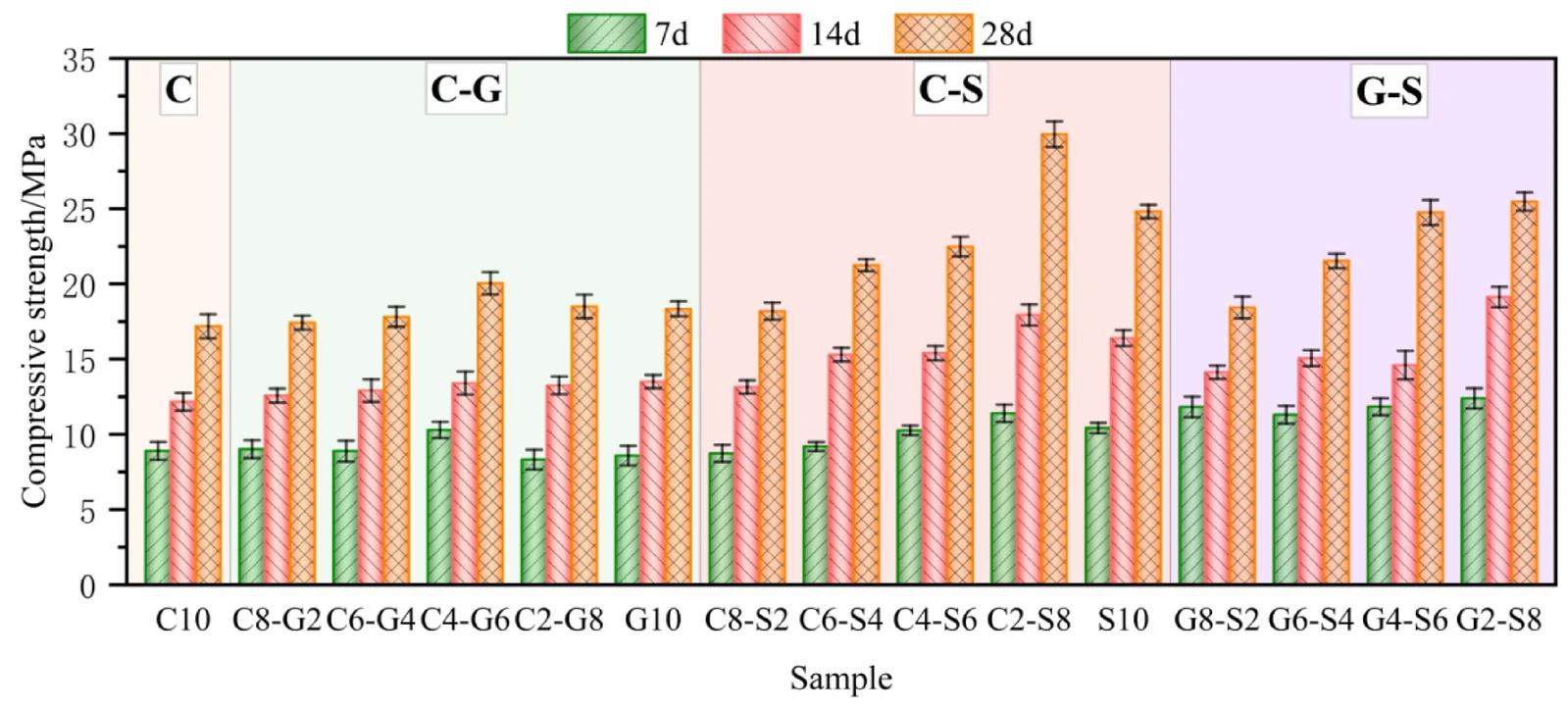
Effect of GGBFS and SF on the compressive strength of UDGCMs. Error bars represent the standard deviation (SD).

**Figure 5:**
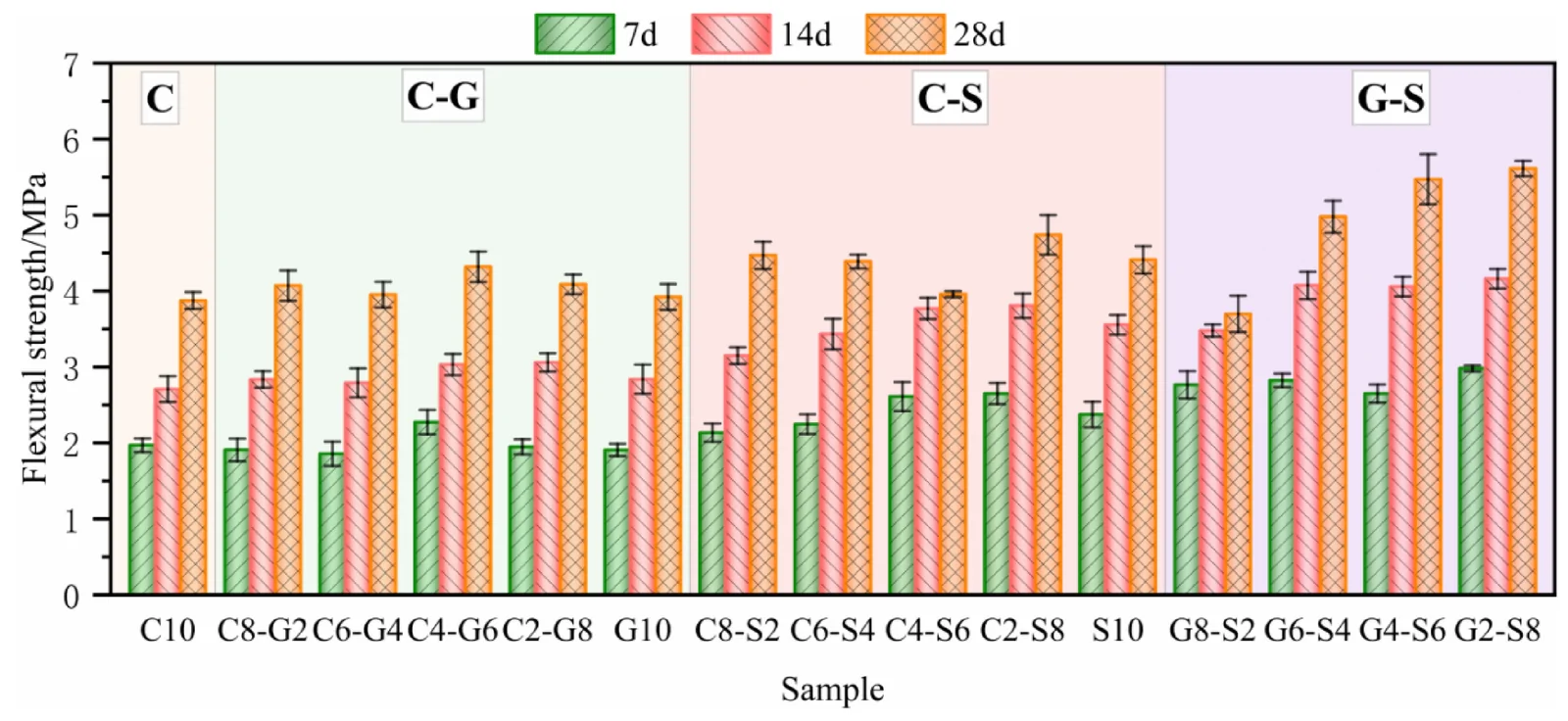
Effect of GGBFS and SF on the flexural strength of UDGCMs. Error bars represent the SD.

**Figure 6:**
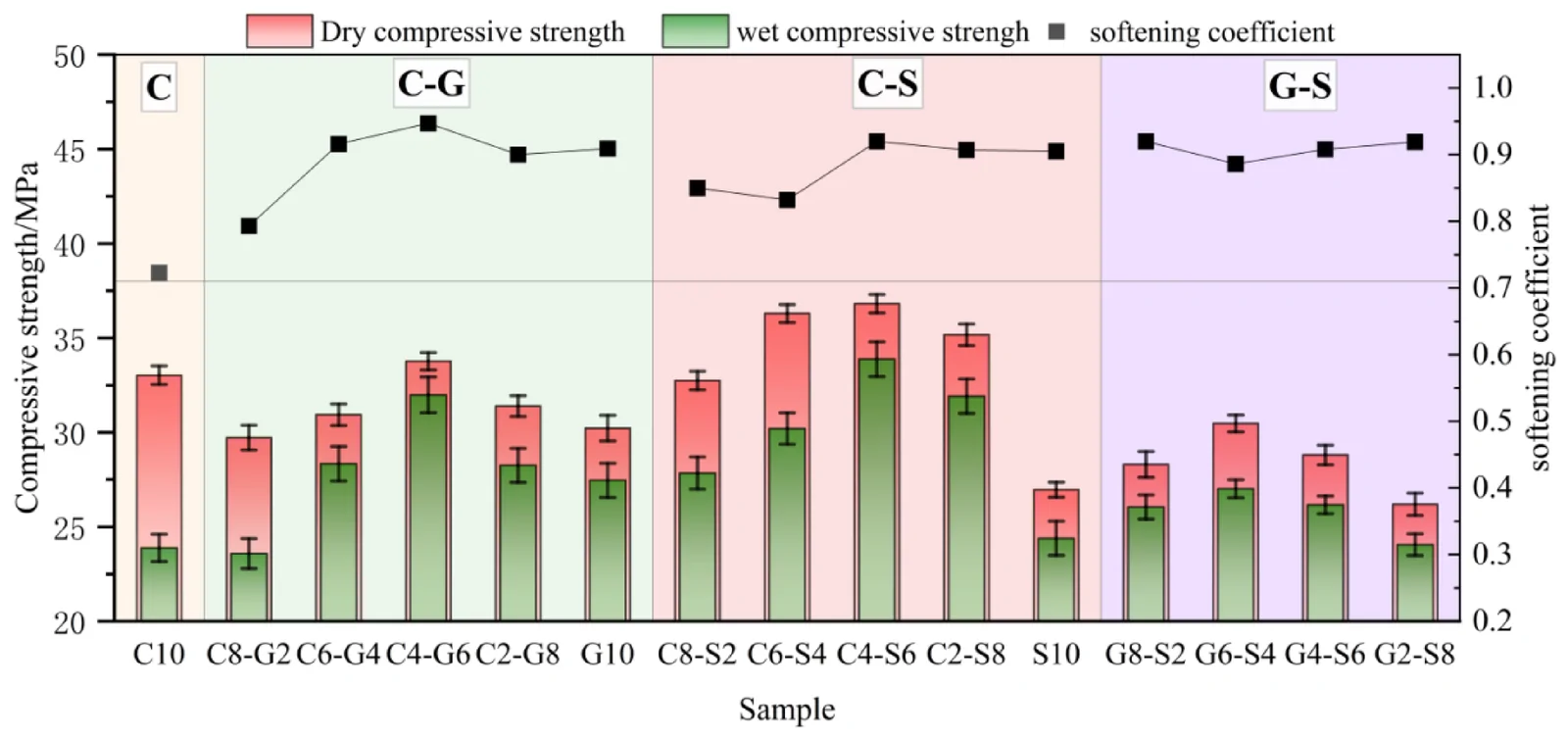
Effect of GGBFS and SF on the softening coefficient of UDGCMs. Error bars represent the SD.

**Figure 7:**
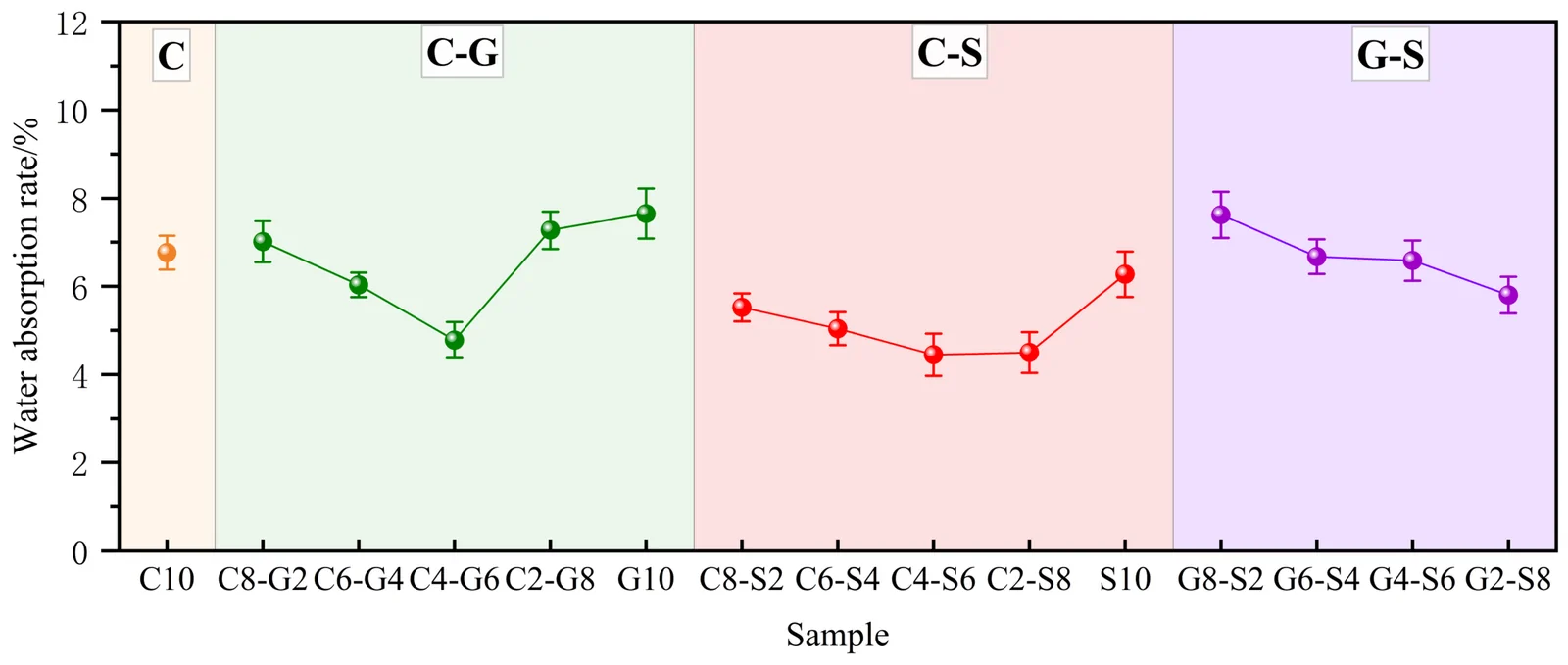
Effect of GGBFS and SF on the water absorption rate of UDGCMs at 24 h. Error bars represent the SD.

**Figure 8:**
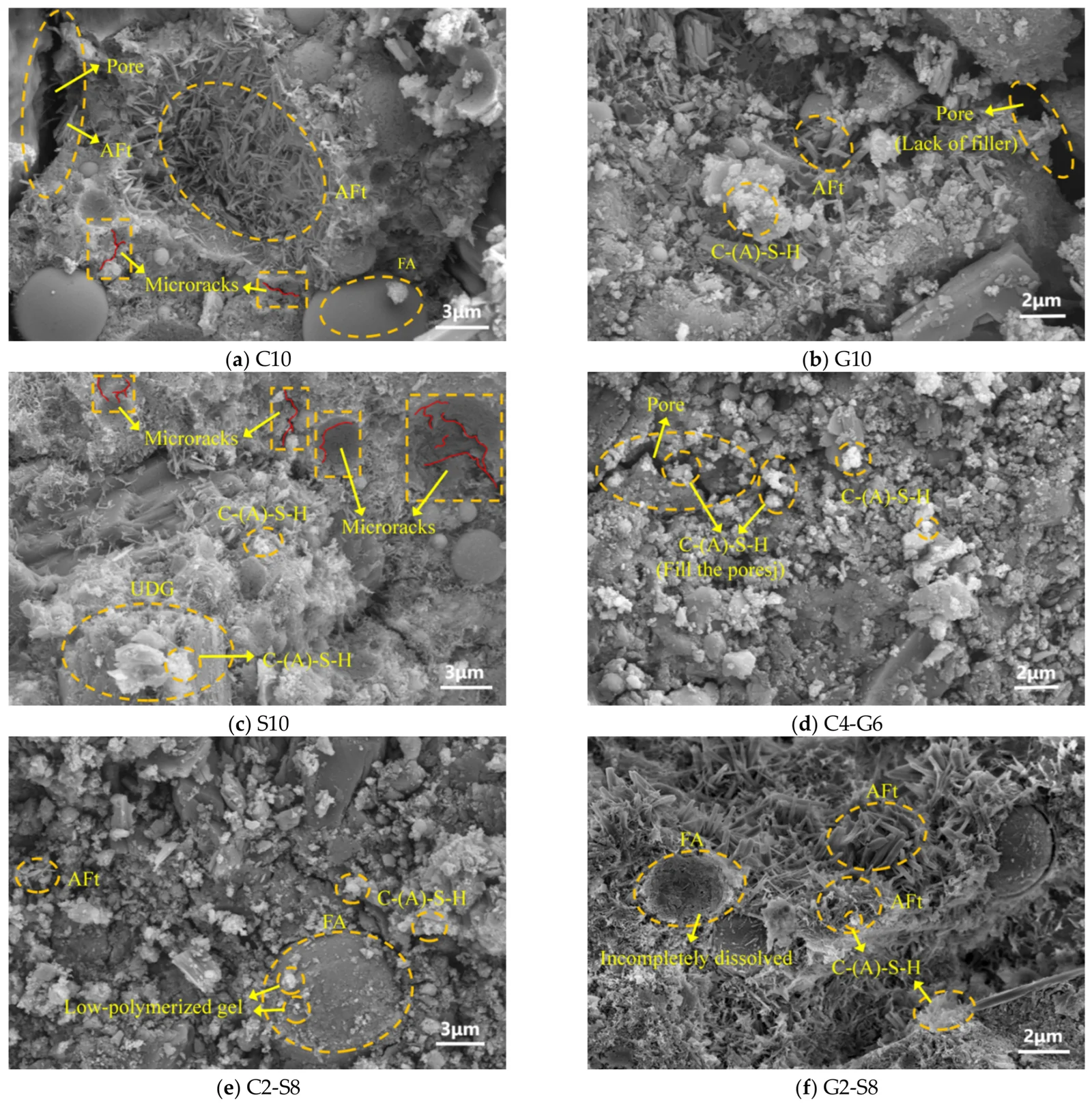
Microscopic morphology of different groups of specimens at 28 days.

**Figure 9:**
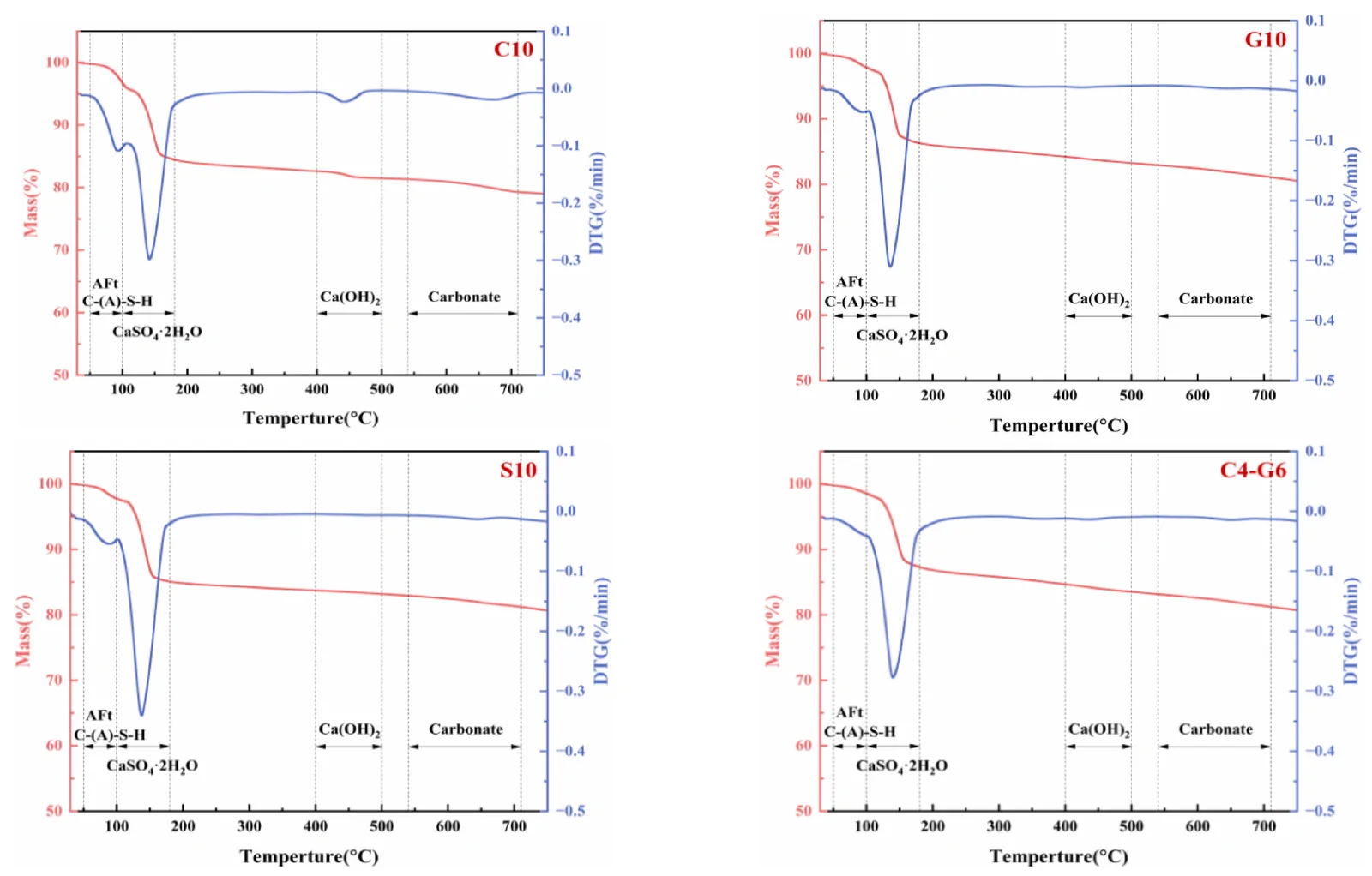

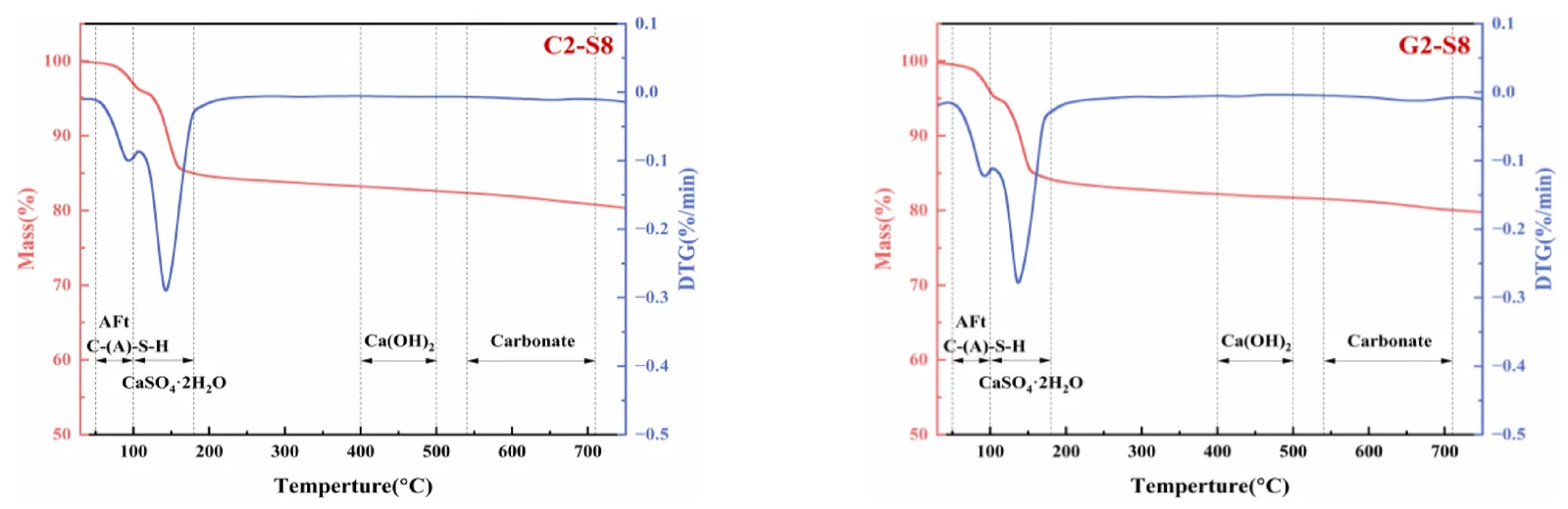
The TG-DTG curves of hydration products from various groups at 28 days.

**Figure 10:**
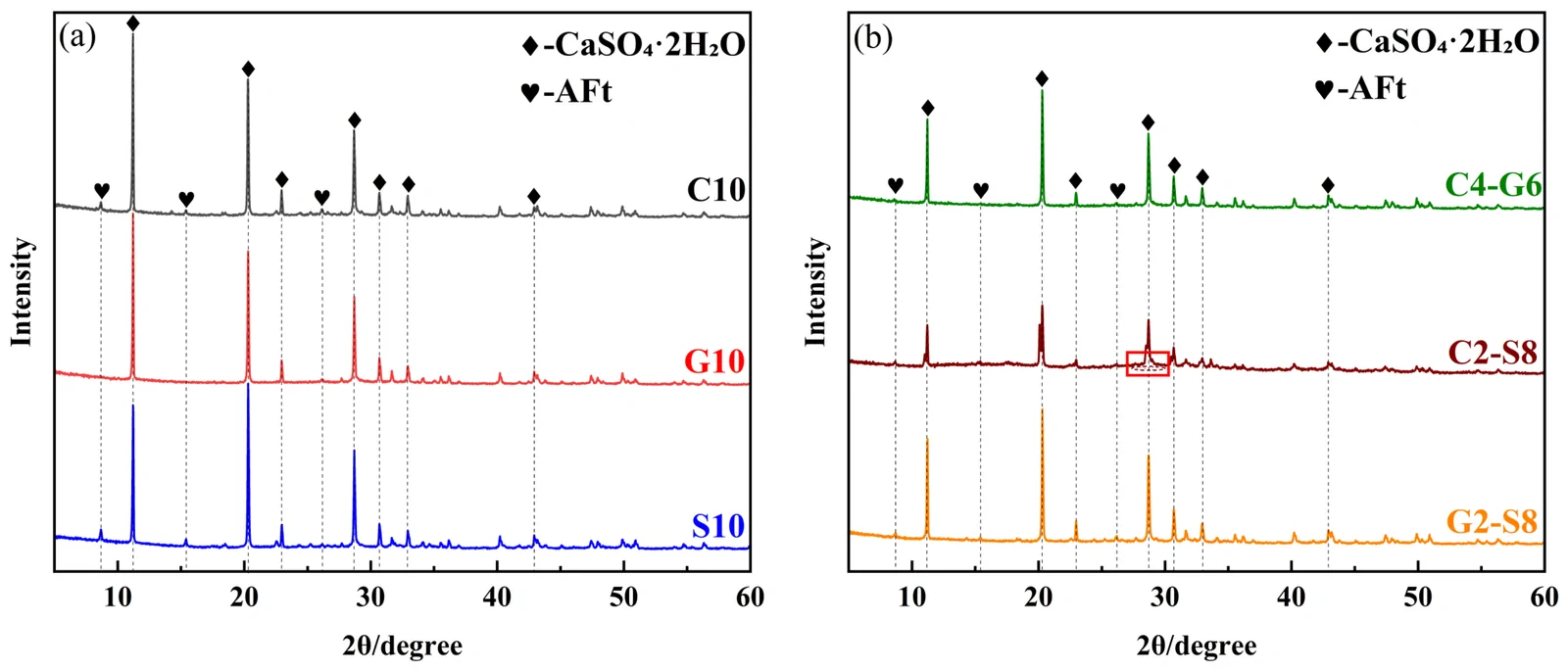
XRD patterns of hydration products of different groups at 28 days. (**a**) C10, G10, and S10 groups; (**b**) C4-G6, C2-S8, and G2-S8 groups.

**Figure 11:**
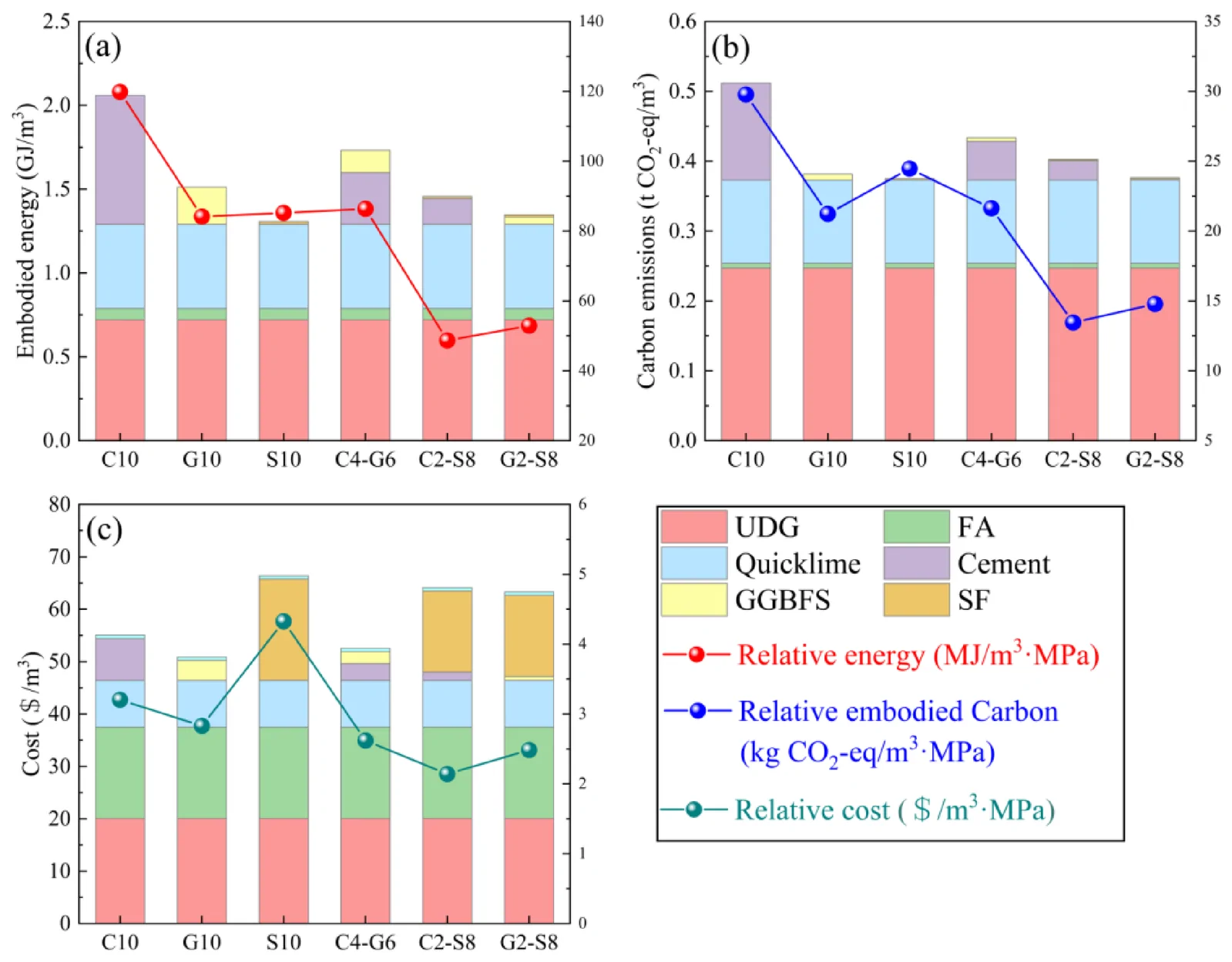
Environmental and economic assessment of UDGCMs. (**a**) Embodied Energy, (**b**) Carbon Emission, (**c**) Cost.

**Table 1:** Main chemical compositions of experimental raw materials w (%).

Raw Materials	SiO_2_	Al_2_O_3_	Fe_2_O_3_	CaO	MgO	SO_3_	Na_2_O	Others
UDG	1.97	0.54	0.14	44.27	0.46	50.74	0.32	1.56
FA	45.68	16.72	10.42	13.37	4.37	1.73	3.18	4.53
Quicklime	1.58	0.47	0.49	93.59	2.36	0.56	0.54	0.41
Cement	18.69	3.95	4.32	65.26	1.52	3.72	0.83	1.71
GGBFS	30.92	8.82	0.49	45.73	6.96	2.56	0.96	3.56
SF	94.33	0.29	0.20	1.94	0.43	1.02	0.24	1.55

**Table 2:** The proportion design of UDGCM (kg/m^3^).

Group	Name	UDG	FA	Quicklime	Cement	GGBFS	SF	Water
C	C10	1003.68	669.12	100.37	167.28	0	0	418.20
C-G	C8-G2	1003.68	669.12	100.37	133.82	33.46	0	418.20
	C6-G4	1003.68	669.12	100.37	100.37	66.91	0	418.20
	C4-G6	1003.68	669.12	100.37	66.91	100.37	0	418.20
	C2-G8	1003.68	669.12	100.37	33.46	133.82	0	418.20
	G10	1003.68	669.12	100.37	0	167.28	0	418.20
C-S	C8-S2	1003.68	669.12	100.37	133.82	0	33.46	418.20
	C6-S4	1003.68	669.12	100.37	100.37	0	66.91	418.20
	C4-S6	1003.68	669.12	100.37	66.91	0	100.37	418.20
	C2-S8	1003.68	669.12	100.37	33.46	0	133.82	418.20
	S10	1003.68	669.12	100.37	0	0	167.28	418.20
G-S	G8-S2	1003.68	669.12	100.37	0	133.82	33.46	418.20
	G6-S4	1003.68	669.12	100.37	0	100.37	66.91	418.20
	G4-S6	1003.68	669.12	100.37	0	66.91	100.37	418.20
	G2-S8	1003.68	669.12	100.37	0	33.46	133.82	418.20

**Table 3:** The mass loss of different groups in each temperature range (wt. %).

Range	C10	G10	S10	C4-G6	C2-S8	G2-S8
50–100/°C	3.05	1.84	2.03	1.23	2.83	2.66
100–180/°C	12.26	11.59	12.70	11.25	11.99	11.83
400–500/°C	1.15	0.97	0.56	1.15	0.62	0.60
540–710/°C	2.04	1.82	1.70	1.91	1.56	1.80

**Table 4:** Raw material environmental and economic analysis data.

Raw Materials	Embodied Energy (MJ/kg)	Carbon Emission (kg CO_2_-eq/kg)	Cost (USD/kg)
UDG [57,58,59]	0.719	0.246	0.02
FA [60]	0.1	0.01	0.026
Cement [61]	4.6	0.83	0.048
Quicklime [59,62]	5	1.19	0.089
GGBFS [63,64]	1.33	0.052	0.023
SF [63,64]	0.10	0.014	0.1156
Water [65]	0	0	0.0015

## Data Availability

The original contributions presented in this study are included in the article. Further inquiries can be directed to the corresponding author.
